# Can short-term memory be trained?

**DOI:** 10.3758/s13421-019-00901-z

**Published:** 2019-02-27

**Authors:** Dennis G. Norris, Jane Hall, Susan E. Gathercole

**Affiliations:** MRC Cognition and Brain Sciences Unit, University of Cambridge, Cambridge, UK

**Keywords:** Short-term memory, Memory training

## Abstract

Is the capacity of short-term memory fixed, or does it improve with practice? It is already known that training on complex working memory tasks is more likely to transfer to untrained tasks with similar properties, but this approach has not been extended to the more basic short-term memory system responsible for verbal serial recall. Here we investigated this with adaptive training algorithms widely applied in working memory training. Serial recall of visually presented digits was found to improve over the course of 20 training sessions, but this improvement did not extend to recall of either spoken digits or visually presented letters. In contrast, training on a nonserial visual short-term memory color change detection task did transfer to a line orientation change detection task. We suggest that training only generates substantial transfer when the unfamiliar demands of the training activities require the development of novel routines that can then be applied to untrained versions of the same paradigm (Gathercole, Dunning, Holmes, & Norris, 2019). In contrast, serial recall of digits is fully supported by the existing verbal short-term memory system and does not require the development of new routines.

**Electronic supplementary material:**

The online version of this article (10.3758/s13421-019-00901-z) contains supplementary material, which is available to authorized users.

In this study, we asked whether the capacity of short-term memory (STM) can be improved with practice. In recent years there has been strong interest in the potential of cognitive training programs to enhance mental capacity (Bavelier, Green, Pouget, & Schrater, 2012; Simons et al., 2016) Many training programs employ complex working memory (WM) activities that combine serial recall of memory sequences with other processing demands. For example, participants may be required to engage in distractor activities interpolated between the presentation of memory items (Chein & Morrison, 2010) or to continuously update the sequence of memory items to be remembered (Dahlin, Neely, Larsson, Backman, & Nyberg, 2008; Jaeggi, Buschkuehl, Jonides, & Perrig, 2008). In these programs, the difficulty of the training task adapts as performance improves with practice. After more than a decade of research in this field, the consensus is that this kind of training generates reliable near transfer to untrained WM tasks with similar task demands. However, there is little far transfer to different activities that are also associated with WM, such as attentional control, reasoning, and learning (Cortese et al., 2015; Melby-Lervåg & Hulme, 2013, 2016; Simons et al., 2016).

To explain this restricted pattern of transfer we have proposed that training on complex WM tasks involves acquiring a new cognitive skill (Gathercole, Dunning, Holmes, & Norris, 2019). The suggestion is that to accomplish the unfamiliar tasks present in most WM training programs, trainees must develop novel routines that coordinate the cognitive processes required. Learning a new routine follows the usual course of the acquisition of any cognitive skill (Anderson, 1982; Taatgen, 2013). With practice, the routine becomes more efficient and less demanding of general cognitive resources, and performance improves. Transfer will only occur if the routine can be successfully applied to a new task, and this will only happen if the task demands are closely matched. Consistent with this framework, a meta-analysis of WM training studies showed that substantial transfer following WM training is largely restricted to cases in which both the trained and untrained tasks share the same complex WM paradigm (Gathercole et al., 2019).

We have made the strong claim that new routines will only be developed if existing mechanisms and processes are not available to support the training activities. If existing mechanisms are available, a new routine is not required, and there will therefore be little transfer. It is proposed that verbal STM measures such as digit span are examples of tasks that do not demand a new routine (Gathercole et al., 2019). Highly specialized processes in verbal STM are responsible for the encoding, maintenance, and retrieval of phonological material in its original sequence, with key phenomena being successfully simulated by computational models incorporating separate item- and order-encoding mechanisms (Hurlstone, Hitch, & Baddeley, 2014). These processes are frequently engaged in everyday situations outside of the laboratory, including learning new words (Baddeley, Gathercole, & Papagno, 1998; Gathercole, 2006), following complex verbal instructions (Engle, Carullo, & Collins, 1991; Jaroslawska, Gathercole, Logie, & Holmes, 2016), and performing mental arithmetic (Adams & Hitch, 1997; Geary, Hoard, Byrd-Craven, & DeSoto, 2004; McLean & Hitch, 1999). On this basis, the cognitive-routine framework predicts that, in contrast to complex WM paradigms, training on verbal STM measures will generate little transfer to other verbal serial-recall tasks.

Surprisingly little research has applied the adaptive computerized algorithms widely employed in WM training studies to more simple verbal STM tasks. Older studies involving small numbers of individuals training on single tasks over extended periods have demonstrated that performance on digit serial recall can improve with practice. In the first study of its kind, two adults received more than 50 sessions of digit span testing spread across a period of four months (Martin & Fernberger, 1929). Span increased by about 40% in both cases, and this was accompanied by reports of grouping strategies. Striking evidence that memory span gains across training are driven by mnemonic strategies was provided by a study by Chase and Ericsson (1981) of S.F., an adult whose digit span increased from seven to 79 items over 2 years of practice. This was achieved by recoding digit sequences into long-distance running times that he was familiar with as a long-distance runner. However, the increase in S.F.’s memory span was entirely restricted to digit sequences. When the memory sequences were composed of letters, his span remained at seven across the full training period.

A second long-distance runner, D.D., was instructed to use the same strategy S.F. had developed, and his span also increased substantially (for a detailed analysis of D.D.’s recall strategies, see Ericsson & Staszewski, 1989; Staszewski, 1990; Yoon, Ericsson, & Donatelli, 2018). Other studies have also demonstrated that digit span expands when participants use other elaborate encoding and retrieval strategies, such as the method of loci, the method of mental imagery, and associations between digit sequences and famous historical dates (Kliegl, Smith, Heckhausen, & Baltes, 1987, and Susukita, 1933, cited in Kliegl et al., 1987). Adopting a rather different approach, Reisberg, Rappaport, and O’Shaughnessy (1984) trained participants to map digits onto finger movements. In this study, digit span increased by up to 50%.

In each of these cases, the increase in span was accompanied by the use of complex mnemonic strategies that combine existing knowledge with either sequences of multiple digits or well-learned sequences that could be used as cues for retrieval. The gains therefore appear to be a consequence of using long-term memory to support the encoding and retrieval of memory items, in conjunction with fixed-capacity verbal STM (Norris, 2017). There is little evidence for fundamental capacity changes in verbal STM with extensive practice.

In the only study of its kind, to our knowledge, Harrison et al. (2013) employed an adaptive computerized STM training regime that consisted of two simple serial tasks—letter span and a spatial span task involving recall of the spatial locations of cells highlighted successively in a matrix. They compared simple span training with two adaptive training programs employing complex span and visual search tasks. Although performance on untrained STM tasks of word span (a verbal serial-recall task) and arrow span (spatial serial recall) improved following training, equivalent benefits were also found for visual search training. There was therefore no selective enhancement of simple memory span by serial-recall training. Other studies have also failed to detect significant transfer of the Cogmed WM training program to digit span, despite its inclusion of a letter span task included in a small number of training sessions (Brehmer, Westerberg, & Bäckman, 2012; Dunning & Holmes, 2014; Gray et al., 2012; Hardy, Willard, Allen, & Bonner, 2013).

In the present study, we tested directly whether the capacity of verbal STM can be enhanced through task-specific training of verbal serial recall. Transfer to untrained STM tasks was compared in three groups, each receiving adaptive training on one of the following STM tasks: digit span, circle span, and color change detection. *Circle span* involves the serial recall of spatial locations highlighted in a sequence at presentation (Minear et al., 2016). Like the dot matrix (Alloway, Gathercole, Kirkwood, & Elliott, 2008), Corsi block (Darling, Della Sala, Logie, & Cantagallo, 2006), and span-board (Wechsler, 1981) tasks, circle span is considered to tap a limited-capacity visuospatial STM system (Logie & Pearson, 1997). The *color change detection* task has been widely used as a measure of the capacity of visual STM (e.g., Awh, Barton, & Vogel, 2007). Developed by Luck and Vogel (1997), it involves participants detecting changes in the colors of individual squares presented simultaneously and briefly in a multi-item visual display. It provides the ideal active-control training condition for the two serial-recall training conditions of digit and circle span, as it does not require the retention of serial order. Performance on this task has already been shown to improve with training. With an adaptive algorithm, Buschkuehl, Jaeggi, Mueller, Shah, and Jonides (2017) reported that set size increased from 6.3 to 8.8 over ten sessions of 300 trials each. In a related nonadaptive task in which only the target square was presented at test, rather than the entire display, Xu, Adam, Fang, and Vogel (2018) reported an increase in memory capacity from 2.1 to 3.0 across 60 days of training. Harrison et al. (2013) reported no changes in change detection following training on two complex span tasks involving serial recall. This suggests that the paradigms tap independent STM and WM systems.

The primary aim of the present study was to investigate what changes occur following digit span training. We set out to track transfer by systematically varying the individual features of the trained tasks in a set of untrained tasks administered before and after training. The untrained verbal serial-recall tasks were spoken digit span (a change in presentation modality from the visual trained task) and visual letter span (a change in verbal category). Substantial experimental evidence has shown that each of these input forms gains ready access to the phonological storage component of verbal STM (Baddeley, Lewis, & Vallar, 1984).

The design of this study allowed us to address several hypotheses regarding the transferability of STM training. In each case, we did this by comparing the training program of interest with the most appropriate active-control program to test each hypothesis. The first hypothesis was that there would be no transfer following digit span training to other verbal serial-recall tasks, because such tasks depend on specialized processes that are already in place in verbal STM (Gathercole et al., 2019). As a consequence, they would not require the development of the novel routines proposed to be the primary source of transfer to untrained tasks with compatible structures. There should therefore be no transfer across verbal serial-recall tasks, even though they place comparable demands on the retention of phonological serial-order information. This should be evident when comparing the impact of digit span training with that of circle span training, the most similar active-control condition, which differed in the domain of STM, but not in the requirement for serial recall. Such an outcome would indicate not only that the fundamental capacity of verbal STM is impervious to training, but also that any substantial on-task training gains are mediated by processes that operate largely outside of STM, such as recoding or chunking (Ericsson, Chase, & Faloon, 1980; Martin & Fernberger, 1929). Later in this article, we speculate that more subtle improvements may nonetheless result from optimization or fine-tuning of the task model to its unique combination of features.

An alternative hypothesis is that verbal STM can be trained. This would be consistent with claims that the cognitive and neural processes that underpin the broader WM system in which STM is embedded can be modified by intensive training (Astle, Barnes, Baker, Colclough, & Woolrich, 2015; Klingberg, 2010). If transfer does extend to untrained serial-recall tasks, the key question is: to which tasks? If the serial-order mechanism is both trainable and specific to verbal STM, transfer should not extend beyond verbal serial-recall tasks to any other untrained tasks, including the circle span measure of spatial STM. In this way, the data have the potential to inform long-standing debate about the extent to which the STM mechanisms for retaining serial order are domain-specific or domain-general (Abrahamse, Van Dijck, Majerus, & Fias, 2014; Alloway, Gathercole, & Pickering, 2006; Bayliss, Jarrold, Gunn, & Baddeley, 2003; Engle et al., 1991; Hanley, Young, & Pearson, 1991; Hurlstone et al., 2014; Majerus et al., 2010). We asked whether transfer is restricted to serial-recall tasks in the same domain (from digit span to letter span and spoken digit span) or extends across domains (from digit span to circle span or circle span to digit span). Here, the critical comparison to evaluate the specificity of transfer would be digit span training versus nonserial color change training.

To further test the limits on transfer, other untrained tasks were included that did not require memory for serial order. Pattern span involves recall of the pattern of filled cells in a static grid. This measure of visual STM involves the recall of filled cells in a grid that are displayed simultaneously and can be recalled in any order. Under some conditions, performance on this test has been found to be dissociable from serial spatial STM tests such as Corsi block recall, possibly reflecting fractionation of visuospatial STM into separate visual and spatial components (Della Sala, Gray, Baddeley, Allamano, & Wilson, 1999). We might therefore anticipate no transfer from either digit span or circle span training (relative to color change detection training) to pattern span.

An untrained line orientation change detection test was also included, in which the array was composed of multiple lines at different orientations and in which participants judged whether the orientation of a single element had changed or remained the same. Alongside color change detection training, this allowed us to test whether training in verbal or spatial serial recall generates benefits that extend to nonserial visual STM. We predicted that it would not, as serial recall appears to reflect a distinct and purely visual system of temporary storage. The inclusion of this training condition also provided an opportunity to explore whether training in one visual change detection task (color change detection) transfers to the ability to detect changes in other visual features in a similar task environment. To date, there has been little research on transfer following training on this paradigm; only a single study has been performed, and this showed no transfer across to variants of the same paradigm with minor changes (Gaspar, Neider, Simons, McCarley, & Kramer, 2013). A finding of positive transfer to orientation change detection following color change detection training would provide preliminary evidence for training-related improvement in the ability to detect mismatches in the properties of visual displays, rather than more specifically to detect changes in the colors of individual objects. In the absence of strong hypotheses regarding the outcome, comparisons were made between the color change training group and each of the other three training groups, including a passive control group that received no training, as well as the digit and circle span training groups, which formed the two active controls.

## Method

### Participants

Eighty English-speaking adults between 18 and 35 years of age were recruited from the Medical Research Council Cognition and Brain Sciences Unit (CBU) Volunteer Panel. Participants received a payment for their time and travel expenses. The study was approved by the University of Cambridge Psychology Research Ethics Committee (CPREC 2014.76). The Participant Information Sheet provided in advance of recruitment outlined a standard hourly payment for participation in the study and reimbursement for travel expenses. It explained that participants would be randomly allocated to conditions, with 10 h of paid home-based iPad training for some but not all individuals. The participants were allocated to training conditions (digit span, circle span, change detection, and no training) on a random basis, subject to the constraint that there were 20 participants in each training condition. This sample size yields power of .91 to detect a large effect size, f^2^ = .35 with a general linear regression model (GLM), and .55 to detect a medium effect size, f^2^ = .35. The sample size was determined on the basis of the outcomes of a meta-analysis of near transfer following WM training reported by Gathercole et al. (2019). On the basis of the limited available evidence, they concluded that, in contrast to the robust transfer found following training on complex WM tasks, gains in verbal STM are at best “small in magnitude and may be reliably detected only under conditions of higher statistical power than the standard WM training study” (p. 20). The present sample size of 20 participants per training group falls within the standard range for WM training studies (Dunning & Holmes, 2014; Harrison et al., 2013; Henry, Messer, & Nash, 2014).

### Procedure

Individuals made two 3-h visits to the CBU. On the first visit, each person was given an iPad with a retina display, for use only in the experiment, that provided access to the training program. Participants first completed eight iPad transfer tasks and then eight tests from the Automated Working Memory Assessment (AWMA). The data from the AWMA are reported for completeness only. The training program was then demonstrated, and participants took the iPad away with them to perform the training. The second visit took place shortly after the final session, approximately three weeks later. Participants again completed the iPad and AWMA transfer tests, and then they returned the iPad. All phases of the experiment apart from administration of the AWMA were presented on iPads with a display resolution of 2,048 × 1,536 pixels in landscape mode. At both the pre- and posttraining sessions, there was also a resting-state magnetoencephalography session, the data from which will be reported elsewhere.

### Transfer

Each of the following tasks was administered at the two visits to the CBU, before and after completing the training program. The eight tasks were presented on the iPad, with common designs and structures where possible. After each session of transfer and training, the data were automatically uploaded to a server at the CBU. Two participants did not complete the pretraining line orientation change detection task, and a further participant did not complete the posttraining line orientation task.

#### Visual digit span

Digits were presented at a rate of one per 750 ms, with each digit being displayed for 500 ms and a blank interval of 250 ms between digits. At the end of the digit sequence, a numeric keyboard (the digits 1–9 in 3 × 3 telephone keypad layout) was displayed, and participants pressed the keys in the order in which the digits had appeared. Below the keyboard was a “Done” key that participants pressed after recall had been completed. With list lengths of nine or less, the digits were sampled randomly without replacement from the digits 1–9. With list lengths greater than nine, the initial set of nine digits was supplemented with a further, randomly sampled *N* digits. No digits appeared twice in succession, and there were no runs of three or more consecutive ascending or descending digits. Testing began with a block of six trials with a list length of four, and increased by one when participants got four or more of the six trials at that length completely correct. Testing continued until participants failed to reach this continuation threshold. Span was determined to be the longest list length for which four or more lists were recalled correctly. Once span had been determined, 12 further trials were presented at each of the lengths span + 1 and span + 2. The measure of performance was 2 * the number of items recalled correctly during span setting + the numbers of items recalled correctly at span + 1 and span + 2. This gave equal weight to trials at each list length.

#### Spoken digit span

This task was identical to the visual digit task, except that the stimuli were digits spoken by a male speaker. The digit sound files were padded out with silence to be 500 ms long. Each digit sound file was followed by 250 ms of silence. The method for determining span and the numbers of trials at each of span + 1 and span + 2 were identical to the methods employed for visual digit span.

#### Letter span

This task was also identical to the visual digit span task, except that the stimulus set was composed of the consonants B, F, H, J, L, M, Q, R, and S. The letters on a 3 × 3 keyboard were arranged in alphabetical order. The method for determining span and the numbers of trials at each of span + 1 and span + 2 were identical to the methods employed for visual digit span.

#### Circle span

Participants were presented with an array of pseudorandomly positioned circles with a radius of 81 pixels and a minimum center-to-center separation of 272 pixels. All circles were colored medium blue on a gray background and then, in random sequence, each circle turned light blue for 250 ms. The rate of presentation was 750 ms per circle. At the end of the sequence, all circles remained visible, and participants were instructed to touch the circles in the order in which they had been displayed. The method for determining span and the numbers of trials at each of span + 1 and span + 2 were identical to the methods employed for visual digit span. Although this task has been used elsewhere as a test of spatial STM (Minear et al., 2016), the possibility cannot be ruled out that participants might attempt to encode the location of the circles verbally. This could introduce an element of verbal STM training in the circle span task. This possibility should be minimized here by the fact that the configuration of the circles varied randomly from trial to trial, making it difficult to assign a set of consistent verbal labels to the locations of the circles.

#### Pattern span

On each trial a 6 × 6 grid was presented for 500 ms. Initially, four of the cells in the grid were displayed in red. After a delay of 1,000 ms, an empty grid was presented, and participants had to touch the squares that had been presented in red. The squares could be touched in any order. The method for determining span and the numbers of trials at each of span + 1 and span + 2 were identical to the methods employed for visual digit span.

#### Color change detection

The procedure was based on that used by Luck and Vogel (1997). Participants saw an array containing between three and 23 colored squares presented for 250 ms. The colors of the squares were chosen at random with replacement from a set of seven readily discriminable colors. The locations of the squares (38 pixels) were random, subject to the constraint that a minimum distance of 117 pixels should separate the centers of the squares.

After a blank retention interval of 1,000 ms, a probe display appeared for 500 ms. The probe was constructed by repeating the previous array, but with one square chosen at random to be the probe square. The color of the probe square either remained the same as in the initial display or, in 50% of the trials, was changed to another randomly chosen color. The location of the probe square was indicated by a red rectangle. Participants had a maximum of 5,000 ms to judge whether the color of the probe square had changed. In all, 20 trials were presented for each of the array sizes 6, 9, and 12. Cowan’s *K* was used to provide a measure of STM capacity for this task (Cowan, 2001; Cowan et al., 2005), where *K* = display size × (proportion hits – proportion false alarms). The mean *K* was computed over the three array sizes, and this measure was used for the purposes of analysis.

#### Direction change detection

The orientation change detection task was identical to the color change detection task, except that the colored squares were replaced by black lines that could appear in one of four orientations (vertical, horizontal, or either diagonal). On each probe trial, one line was cued by a red circle, with a 50% probability that the orientation of the line would have changed. The mean *K* was computed, as in the color change detection task. Due to technical problems, there were incomplete data on this transfer task for two participants in the circle-training condition. Their data were omitted from the reported analyses.

#### Automated Working Memory Assessment

The following tests from the AWMA (Alloway, 2007), a standardized test battery of STM and WM tests, were administered on a desktop PC. Each employed a span procedure. The tests were word span and nonword span tests (verbal STM), dot matrix and mazes memory (visuospatial STM), listening span and counting span (verbal WM), and Mr X and spatial span (visuospatial WM). The analysis was based on raw scores. It should be noted that these tasks shared fewer presentational and task features in common with the most closely matched training activities than did the iPad transfer tasks. On this basis, no strong predictions could be made regarding the impact and potential specificity of the training conditions on these transfer tests. For completeness, the data and statistical outcomes are reported in the supplemental material.

#### Matrix reasoning

This test of nonverbal reasoning from the Wechsler Adult Intelligence Scales involves selecting the missing part to complete visuospatial patterns. Raw scores were used for the purpose of analysis.

### Training

Each participant completed either digit span, circle span, color change detection training, or no training. The participants in the three active training conditions were asked to complete 20 sessions in total on their iPad program. The maximum time allowed for completion of a session was 40 min, with no more than three sessions per day and an interval of no more than two days between successive training sessions. Training could only be performed between 7 a.m. and 11 p.m.

#### Digit span training

Each training session consisted of eight blocks of ten trials employing the same procedure as the visual digit span transfer test, with the exception that set size was varied adaptively. Training began with a sequence of three digits, and increased by one when participants got eight or more trials completely correct in a block. The length of the sequence decreased by one if participants got two or fewer trials correct. Due to technical problems, the data for one participant in the digit training condition were lost for the 12th session. The missing score was replaced by the mean score from the 11th and 13th sessions. On average, participants completed the training sessions in in 13.8 days (min 10, max 16, *SD* 1.8). The principal score for the purposes of analysis was the span reached in the final block of each session.

#### Circle span training

Each training session consisted of eight blocks of ten trials employing the same procedure as the circle span transfer task. Training began with a display of three circles and increased by one when participants got eight or more trials completely correct in a block. The number of circles decreased by one if participants got two or fewer trials correct. On average, participants completed the training sessions in 13.25 days (min 10, max 15, *SD* 1.55). The principal score for the purposes of analysis was the span reached in the final block of each session.

#### Color change detection training

In each training session there were eight blocks of 30 trials, employing the same presentation procedure as in the color change detection transfer task. The size of the array was increased by one if participants got 27 or more trials correct, and decreased by one if they got 18 or fewer correct. On average, participants completed the training sessions in 13 days (min 10, max 15, *SD* 1). Two measures derived from each session were used for the purposes of analysis: The first was the capacity measure *K* (Cowan, 2001; Cowan et al., 2005), and the second was the difficulty level (set size) reached by the end of each block.

### Analysis plan

The training and transfer data were analyzed using both traditional null-hypothesis significance testing (NHST) methods and corresponding Bayesian methods. This allowed us to quantify the strength of evidence both in favor of the null hypotheses of the absence of training/transfer effects, and in favor of the alternative hypothesis that there were positive effects. The Bayesian analyses were conducted using JASP (JASP Team, 2015). Bayes factors (BF_10_) were interpreted as follows (Jeffreys, 1961): BFs < 0.33 provide evidence for the null hypothesis; BFs 0.33–3 provide equivocal evidence for both hypotheses; BFs > 3 provide evidence favoring the alternative hypothesis; BFs > 10 and < 0.01 are considered strong evidence in either direction; and BFs > 100 and < 0.001 provide decisive evidence in either direction.

Training effects for the three active training conditions were analyzed in one-way analyses of variance (ANOVAs) with session as the independent variable. Interactions between training conditions and trials were not computed, due to the different performance metrics used for the span and color change detection tasks. For all training conditions, the metric was the difficulty level achieved by each participant in the final block of each session. For digit and circle span, this was the number of items in the sequence, and for color change detection, it was *K*. Both Bayesian and non-Bayesian ANOVAs were performed.

To evaluate the specificity of transfer following training, Bayesian and non-Bayesian linear regression analyses were performed for each combination of the training task of interest and each transfer test. The posttraining transfer measure was the dependent variable, and the pretraining measure and the particular training group contrast were entered as dependent variables in each case. Four group contrasts were made for each transfer measure. Three comparisons contrasted pairs of active adaptive training conditions: digit span versus circle span (testing the domain specificity of serial-recall training), digit span versus color change detection (testing the specificity of serial-recall training), and circle span versus color change detection (testing the specificity of change detection training). A final contrast compared color change detection training with no training, as a test of whether there was transfer across serial and nonserial STM paradigms. For the NHST, a Bonferroni correction was applied on a family-wise basis for the transfer tests, yielding an *α* of .007. For each group contrast and transfer measure combination, initial linear regression analyses were run testing for interactions between pretraining scores and group. Where these were considered to be significant or to favor the alternative hypothesis (*p* < .05 or BF > 3), the group term reported here is taken from the analysis that included the interaction term. If the interaction terms did not meet these criteria, the model was rerun excluding the interaction term, and the group term from this analysis is reported.

## Results

### Training data

**Fig. 1:**
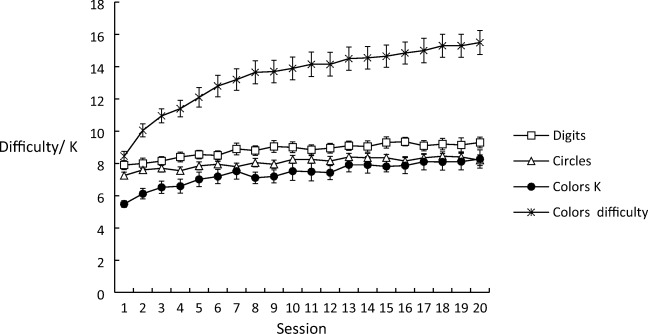
Scores on the final block of each session as a function of training condition and measure

The mean scores achieved at the end of each training session (span for digit and circle training, and both capacity *K* and difficulty level for color change detection) are shown in Fig. 1. Gains across training sessions were considerably greater for color change detection than for either digit span or circle span training. Performance increased from the first to the final training session, by 18% for digit span training and by 13% for circle span training. For color change detection training, the increase was 51% for the capacity measure *K*, and 83% for the difficulty level. This reflects an increase in the number of elements in the array from 8.45 to 15.50.

Bayesian and non-Bayesian one-way ANOVAs were performed on the scores in each session for each training condition. In each case, performance increased significantly across training. For NHST, these results were: digit span, *F*(19, 361) = 7.691, *MSE* = .497, *p* < .001; circle span, *F*(19, 361) = 6.275, *MSE* = .358, *p* < .001; color change detection *K*, *F*(19, 361) = 8.774, *MSE* = 1.227, *p* < .001; color change detection difficulty, *F*(19, 361) = 50.574, *MSE* = 1.447, *p* < .001. Tested against the null model, the BF_10_ values were > 100 for digit span, circle span, color change detection K, and color change detection difficulty level. These outcomes provide decisive evidence that performance improved with training on each task and for each measure.

### Transfer data

Descriptive statistics and analyses of the transfer measures are shown in Table 1. Our first question was whether there is domain-specific transfer to other verbal span tasks following digit span training. This was addressed by comparing posttraining scores on the untrained verbal span tests for the digit and circle span training groups. Unsurprisingly, digit span led to a substantially greater enhancement of digit span performance than did circle span training, according to both NHST and Bayesian analysis. This represents a further demonstration of on-task training. For the two untrained verbal span measures, the evidence for a selective advantage following digit span training was weak. For spoken digit span (a change in the modality of the memory items), the NHST was nonsignificant, and the Bayesian outcome was equivocal, weakly favoring the null hypothesis. For letter span, too, the *p* value was nonsignificant and the Bayes factor value equivocal.

**Table 1:** Descriptive statistics and analyses of the transfer measures, by training group

Transfer Test	Training Group	Pretraining		Posttraining		Effect Size	GLM Group *p*				Bayes Factor_10_			
		*M*	*SD*	*M*	*SD*		Digits vs. Colors	Digits vs. Circles	Colors vs. No Training	Circles vs. Colors	Digits vs. Colors	Digits vs. Circles	Colors vs. No Training	Circles vs. Colors
Visual digit span	Digits	406.40	118.97	658.55	197.14	1.60	< .001	< .001	.070	.378	> 100	88.880	0.746	0.265
	Circles	405.05	143.19	473.55	143.94	0.48								
	Color	410.15	164.83	450.40	134.36	0.27								
	None	438.40	156.32	421.55	143.43	– 0.11								
Spoken digit span	Digits	443.20	129.29	530.40	150.29	0.62	.044	.275	.796	.358	1.477	0.416	0.240	0.325
	Circles	470.15	144.98	508.40	141.50	0.27								
	Color	430.85	112.75	452.40	120.33	0.18								
	None	431.20	139.43	444.15	144.92	0.09								
Letter span	Digits	330.55	108.86	404.00	143.97	0.58	.300	.040	.280	.254	0.244	1.184	0.266	0.286
	Circles	320.20	117.79	332.85	149.94	0.09								
	Color	275.55	102.88	318.65	128.99	0.37								
	None	309.05	110.44	324.85	144.86	0.12								
Circle span	Digits	345.80	77.21	414.30	84.39	0.85	.106	< .001	< .001	< .001	0.694	> 100	22.94	> 100
	Circles	380.30	95.12	601.60	153.98	1.78								
	Color	318.35	58.89	359.10	80.38	0.59								
	None	353.90	103.96	323.15	110.33	– 0.29								
Pattern span	Digits	396.80	215.11	460.05	210.14	0.30	.527	.985	.964	.316	0.385	0.265	0.355	0.491
	Circles	445.15	214.16	485.50	194.37	0.20								
	Color	352.10	156.30	403.45	178.05	0.31								
	None	341.15	167.56	396.55	168.42	0.33								
Color detection	Digits	4.99	1.96	5.03	1.67	0.02	< .001	.162	< .001	< .001	> 100	0.539	> 100	> 100
	Circles	5.28	1.41	5.73	1.37	0.32								
	Color	4.94	1.32	7.34	0.78	2.28								
	None	5.26	1.69	5.71	2.15	0.23								
Orientation detection	Digits	3.34	1.83	4.33	2.67	0.44	.217	.412	< .001	< .001	0.813	0.262	> 100	9.259
	Circles	3.72	1.95	4.30	1.95	0.30								
	Color	3.99	1.74	5.87	1.80	1.06								
	None	3.13	2.29	3.47	1.96	0.71								

The second question was whether there are domain-general benefits to serial-recall training. This was addressed in two ways. The first was by comparing the digit span and color change detection training groups on the circle span transfer measure. There was no substantial evidence of transfer: The *p* value was nonsignificant, and the Bayes factor was equivocal, weakly favoring the null hypothesis. The second comparison was between circle span and color change detection training for the three verbal span measures. In each case, the NHST was nonsignificant and the Bayes factor value substantially favored the null hypothesis. We therefore found no substantial evidence for cross-domain transfer across serial-recall tasks in either direction.

The third question was whether there were paradigm-general benefits to training that extended across all three STM training and transfer tasks. This was addressed by comparing the posttraining performance of the color change detection and no-training groups on the five transfer tests that did not involve change detection. It should be noted that this comparison between an active-training and a no-contact control condition was likely to overestimate any potential benefits, and therefore increase the likelihood of a false-positive result (Simons et al., 2016). For two of the three verbal span measures (spoken digit span and letter span), the analyses provided no evidence of training benefits, with nonsignificant *p* values and Bayes factor values showing substantial support for the null hypothesis. For visual digit span, the *p* value was nonsignificant, and the Bayes factor was equivocal and mildly favored the null hypothesis. For circle span, though, we did find evidence of strong transfer, by both NHST and Bayesian analysis. This provided unexpected evidence for transfer from visual STM training to visuospatial serial recall. However, it is notable that there was no evidence for a symmetrical pattern of transfer from circle span training to the color change detection task: With circle training, capacity *K* increased from 5.28 to 5.73. For the no-training condition, a similar increase from 5.26 to 5.71 was observed.

The final question was whether color change detection training generates benefits for a line detection task employing the same paradigm. Here the statistical outcomes were clear. Relative to circle span training, color change detection training was associated with greater improvements in the untrained line orientation change detection task, as indicated by a strongly significant *p* value and a Bayes factor substantially favoring the alternative hypothesis.

## Discussion

Performance improved on all three STM tasks across the course of training. The performance gains across training were relatively small for the two serial-recall tasks—15% for digit span and 12% for circle span training. For color change detection training, the increases were considerably greater, with an estimated STM capacity increase of 51%, and an 83% increase in the size of the array by the end of training.

We observed no positive evidence for transfer from digit span training to circle span, or vice versa. Neither was there strong evidence that digit span training benefited performance on the untrained verbal serial-recall tests of spoken digit span or letter span. The lack of transfer across verbal serial tasks is consistent with the predictions of the cognitive-routine framework (Gathercole et al., 2019). According to this theory, transfer occurs only when the demands of the training tasks cannot readily be met by existing STM mechanisms and processes. Only under these conditions will participants need to develop new cognitive routines that control and coordinate the processes involved in performing the task. When training involves only simple verbal serial-recall tasks, no new routines are required because a well-established and highly practiced set of mechanisms is already in place within verbal STM. There should therefore be no substantial transfer, as we indeed found. As was noted by Gathercole et al. (2019), the exception to this would be individuals with an underdeveloped verbal STM system. In children who do not yet rehearse, rehearsal training does indeed increase memory span (Broadley & MacDonald, 1993; Johnston, Johnson, & Gray, 1987).

The absence of transfer from either serial-recall training program to untrained serial-recall tasks therefore provides no evidence that the capacity of STM can be expanded with intensive training. This is particularly noteworthy for digit span, as the transfer tasks were distinguished only by a single feature—input modality for spoken digit span (visual to auditory), and semantic category for letter span (digits to letters). According to the current understanding of verbal STM, each of these three stimulus forms should be equally readily represented in verbal STM (Baddeley et al., 1984). If training had acted to increase STM capacity, the benefits of this extra capacity should therefore extend to both tasks. One possibility is that training on a single task allows participants to develop category-specific complex recoding strategies that reduce memory load by permit chunking of multi-item sequences (Ericsson et al., 1980; Martin & Fernberger, 1929). Although this hypothesis may explain the corresponding lack of transfer to letter span, it is not consistent with the corresponding absence of transfer to spoken digit span. With equal access to phonological storage for visual and auditory inputs, any beneficial effects of digit-specific encoding strategies would be expected to extend to digits presented in either modality.

Training-induced changes therefore appear to be tied to the semantic category and input modality of the memory items, as well as to paradigm. Why, then, should performance on the trained task improve at all if, as the absence of transfer suggests, the capacity of verbal STM is unchanged? The present data showed a relatively modest increase of 18% in digit span with training. This is considerably smaller that the gains observed in studies that had explicitly trained digit span strategies involving recoding (Ericsson & Staszewski, 1989; Kliegl et al., 1987; Reisberg et al., 1984; Yoon et al., 2018). Moreover, the gains that we found in digit span in the present study appear to be tied to the specific conjunction of the trained task features. One way of explaining this is that extensive training on a single task in which all parameters are fixed (e.g., perceptual, timing, and categorical) allows participants to fine-tune their task model. This could be conceived as a form of learning that takes place within the established system of verbal STM. If performance is finely tuned to all features of a single task, even superficial deviations from the trained task might be sufficient to render the model suboptimal. In this way, subtle changes within an existing system could be detectable in training effects when all task features are preserved, but not generalize to other variants of the same paradigm.

Training on the color change detection measure of visual STM generated both substantial on-task training gains and transfer to another task, in which participants detected changes in the orientation of lines in a multi-item array. To our knowledge, this is the first time that training-induced change has been demonstrated for static visual STM, a resource-limited memory system that has been extensively investigated in recent years (e.g., Alvarez & Cavanagh, 2004; Bays, Catalao, & Husain, 2009). There are several possible explanations for this outcome. Applying the rationale extended to findings of near transfer following WM training (Dahlin et al., 2008; Jaeggi et al., 2008; Klingberg, 2010), it could be interpreted as reflecting genuine plasticity in the capacity of visual STM. An alternative possibility is that the change detection paradigm may require the establishment of a new cognitive routine (Gathercole et al., 2019), and that this is the source of transfer. The very brief presentation of displays containing highly similar objects for a binary change detection judgment certainly imposes highly unfamiliar cognitive demands that quite plausibly might not be met solely through the processes in place within visual STM. The relatively large magnitude of the training gains seen in this task is certainly consistent with mediated learning. Perhaps, then, trainees develop a change detection routine to optimize their performance that—unlike the highly specific tuning to the specific task features seen in digit span training, which shows no substantial generalization—can be readily adapted to the untrained orientation detection task with its very similar demands.

Alternatively, transfer across change detection tasks reflects learning by the participant about the statistical properties of the displays. Such learning might underpin the robust training and transfer gains found for change detection tasks. Orhan and Jacobs (2014) have suggested that the apparent capacity limitations in visual STM might be due to a mismatch between the participant’s internal model and the true statistics of the stimuli. For example, our change detection tasks had a statistical structure of the elements within the display: Colors were not positioned at random but were constrained to have a minimal separation. This became even more constraining as the number of stimuli in the display increased. When the maximum number of items were in the display, they were closely packed, and there was much less room for variation in position than with fewer items. The orientation change task had the same statistical properties. Perhaps, then, learning about the statistics of the displays in one task could readily transfer to the other. An important question as yet unanswered is whether transfer to other change detection tasks would persist if the statistics of the displays changed between the trained and untrained activities.

Participants might also learn about the characteristics of their internal representations of stimuli in change detection tasks. To optimize the readout of information from memory, participants need to have an accurate model of the internal representation that will be produced by a particular input. In Bayesian terms, they need to develop an accurate generative model of the task. This form of learning or adaptation is likely to be tied to the low-level perceptual properties of the stimuli. In the case of serial recall with letters or digits, this might involve nothing more than fine-tuning, but for a completely novel task like change detection, more work might need to be done.

In summary, on-task performance improves after extensive practice with serial recall of visually presented digits. However, there is little evidence that this improvement confers any advantage to recall of visually presented letters, auditorily presented digits, or sequences of spatial locations. Changing either the stimulus domain, the presentation modality, or the category of the memory items eliminated the benefits of training. Digit span training does not substantially improve the capacity of verbal STM. In contrast, training on an unfamiliar color change detection task produces large gains in performance that transfer to a line orientation change detection task. The large improvement in change detection was unexpected, as change detection is often used to estimate core visual STM capacity. This might have been a consequence of learning how to perform a novel task, in much the same way as for more complex WM tasks, and also how to optimize performance by exploiting the statistical properties of the displays.

## Electronic supplementary material

ESM 1 (DOCX 18 kb)
