# Supplementary material for: Can short-term memory be trained?
Source: Mem Cognit. 2019 Feb 27;47(5):1012–23. doi: 10.3758/s13421-019-00901-z (PMC6647533; doi:10.3758/s13421-019-00901-z)
Supplement: Supplementary file 1 — (DOCX 18 kb) [file 13421_2019_901_MOESM1_ESM.docx]

| Supplementary materials. AWMA transfer measures by training group | | | | | | | |  |  |  |  |  |  |  |
| --- | --- | --- | --- | --- | --- | --- | --- | --- | --- | --- | --- | --- | --- | --- |
|  |  | Pre | | Post | |  | GLM group *p* | | | | Bayes Factor_10_ | | | |
| Transfer measures | Training group | M | SD | M | SD | Effect size | Digits vs colours | Digits vs circles | Colours vs no training | Circles vs colours | Digits vs colours | Digits vs circles | Colours vs control | Circles vs colours |
| Word span | Digits | 31.90 | 3.68 | 32.85 | 3.10 | 0.14 | 0.431 | 0.988 | 0.401 | 0.503 | 0.224 | 0.231 | 0.233 | 0.258 |
|  | Circles | 32.20 | 3.41 | 33.05 | 3.30 | 0.13 |  |  |  |  |  |  |  |  |
|  | Colour | 31.55 | 3.59 | 33.15 | 3.88 | 0.21 |  |  |  |  |  |  |  |  |
|  | None | 30.90 | 3.71 | 31.95 | 3.83 | 0.14 |  |  |  |  |  |  |  |  |
| Nonword span | Digits | 19.35 | 3.23 | 20.80 | 3.79 | 0.21 | 0.311 | 0.023 | 0.324 | 0.292 | 0.330 | 2.035 | 0.334 | 0.376 |
|  | Circles | 20.15 | 2.83 | 19.90 | 2.61 | -0.05 |  |  |  |  |  |  |  |  |
|  | Colour | 19.70 | 2.60 | 20.30 | 2.85 | 0.11 |  |  |  |  |  |  |  |  |
|  | None | 18.65 | 3.31 | 18.90 | 3.02 | 0.04 |  |  |  |  |  |  |  |  |
| Listening span | Digits | 20.95 | 3.68 | 23.05 | 3.30 | 0.30 | 0.786 | 0.837 | 0.833 | 0.927 | 0.306 | 0.241 | 0.247 | 0.254 |
|  | Circles | 20.60 | 3.90 | 22.60 | 4.74 | 0.23 |  |  |  |  |  |  |  |  |
|  | Colour | 18.95 | 4.38 | 21.70 | 4.86 | 0.30 |  |  |  |  |  |  |  |  |
|  | None | 19.50 | 5.61 | 22.20 | 6.08 | 0.23 |  |  |  |  |  |  |  |  |
| Counting span | Digits | 28.15 | 3.69 | 28.45 | 4.56 | 0.04 | 0.108 | 0.427 | 0.017 | 0.407 | 0.643 | 0.251 | 2.685 | 0.274 |
|  | Circles | 27.75 | 3.63 | 28.90 | 5.15 | 0.13 |  |  |  |  |  |  |  |  |
|  | Colour | 25.45 | 3.59 | 27.70 | 4.22 | 0.29 |  |  |  |  |  |  |  |  |
|  | None | 27.15 | 5.21 | 26.50 | 6.31 | -0.06 |  |  |  |  |  |  |  |  |
| Dot matrix | Digits | 33.35 | 3.99 | 34.85 | 5.43 | 0.16 | 0.638 | 0.204 | 0.033 | 0.394 | 0.208 | 0.372 | 0.463 | 0.227 |
|  | Circles | 34.90 | 6.90 | 38.00 | 7.48 | 0.22 |  |  |  |  |  |  |  |  |
|  | Colour | 31.95 | 3.98 | 33.95 | 6.30 | 0.19 |  |  |  |  |  |  |  |  |
|  | None | 32.95 | 5.81 | 32.95 | 5.15 | 0.00 |  |  |  |  |  |  |  |  |
| Mazes memory | Digits | 30.35 | 2.82 | 28.85 | 3.03 | -0.26 | 0.200 | 0.150 | 0.120 | 0.814 | 0.794 | 0.857 | 0.824 | 0.378 |
|  | Circles | 31.70 | 3.77 | 30.70 | 3.28 | -0.14 |  |  |  |  |  |  |  |  |
|  | Colour | 29.75 | 2.83 | 29.90 | 2.67 | 0.03 |  |  |  |  |  |  |  |  |
|  | None | 30.50 | 4.90 | 28.85 | 3.79 | -0.19 |  |  |  |  |  |  |  |  |
| Supplementary materials. AWMA transfer measures by training group continued | | | | | | | | | |  |  |  |  |  |
| Mr X | Digits | 21.20 | 5.69 | 24.50 | 6.82 | 0.26 | 0.516 | 0.127 | 0.344 | 0.013 | 0.347 | 0.864 | 0.294 | 4.387 |
|  | Circles | 24.05 | 5.77 | 28.95 | 6.31 | 0.41 |  |  |  |  |  |  |  |  |
|  | Colour | 22.10 | 5.92 | 23.95 | 4.70 | 0.17 |  |  |  |  |  |  |  |  |
|  | None | 21.75 | 8.10 | 22.45 | 7.16 | 0.05 |  |  |  |  |  |  |  |  |
| Spatial span | Digits | 30.30 | 7.01 | 31.15 | 4.90 | 0.07 | 0.354 | 0.022 | 0.220 | 0.306 | 0.311 | 2.741 | 0.335 | 0.287 |
|  | Circles | 31.10 | 5.65 | 34.85 | 5.78 | 0.33 |  |  |  |  |  |  |  |  |
|  | Colour | 27.10 | 7.08 | 30.60 | 6.44 | 0.26 |  |  |  |  |  |  |  |  |
|  | None | 26.65 | 8.11 | 28.50 | 7.74 | 0.12 |  |  |  |  |  |  |  |  |
| Matrix reasoning | Digits | 23.15 | 3.80 | 24.30 | 2.23 | 0.19 | 0.442 | 0.729 | 0.483 | 0.703 | 0.309 | 0.267 | 0.356 | 0.324 |
|  | Circles | 23.15 | 2.56 | 24.10 | 2.27 | 0.20 |  |  |  |  |  |  |  |  |
|  | Colour | 23.65 | 3.23 | 24.05 | 2.74 | 0.07 |  |  |  |  |  |  |  |  |
|  | None | 23.40 | 2.26 | 24.40 | 2.14 | 0.23 |  |  |  |  |  |  |  |  |
